# Comparison of Preoperative Temporal Bone CT with Intraoperative Findings in Patients with Cholesteatoma

**Published:** 2014-01

**Authors:** Mehrdad Rogha, Sayyed Mostafa Hashemi, Farhad Mokhtarinejad, Afrooz Eshaghian, Alireza Dadgostar

**Affiliations:** 1 *Department of Otorhinolaryngology, Kashani Hospital, Isfahan University of Medical Sciences, Isfahan, Iran.*; 2 *Department of Otorhinolaryngology, Loghman Hospital, Shahid Beheshti University of Medical Sciences, Isfahan, Iran.*

**Keywords:** Cholesteatoma, Computed Tomography (CT), Middle Ear

## Abstract

**Introduction:**

Cholesteatoma is traditionally diagnosed by otoscopic examination and treated by surgery. The necessity for imaging in an uncomplicated case is controversial. This study was planned to investigate the usefulness of a preoperative high-resolution computed tomography (HRCT) scan in depicting the status of middle ear structures in the presence of cholesteatoma and also to compare the correspondence between pre- and intraoperative CT findings in patients with cholesteatoma.

**Materials and Methods:**

This prospective descriptive study was performed from January 2009 to May 2011 in 36 patients with cholesteatoma who were referred to the Kashani and Al-Zahra Clinics of Otolaryngology. Preoperative high-resolution temporal bone CT scans (axial and coronal views) were carried out and compared with intraoperative findings.

**Results:**

Evaluation of 36 patients and their CT scans revealed excellent correlation for sigmoid plate erosion, widening of aditus, and erosion of scutum; good correlation for erosion of malleus and tegmen; moderate correlation for lateral canal fistula (LCF) and erosion of mastoid air cells; and poor correlation for facial nerve dehiscence (FND), incus, and stapes erosion.

**Conclusion:**

A preoperative CT scan may be helpful in relation to diagnosis and decision making for surgery in cases of cholesteatoma and ossicular erosion. The CT scan can accurately predict the extent of disease and is helpful for detection of lateral canal fistula, erosions of dural plate, and ossicular erosions. However it is not able to distinguish between cholesteatoma and mucosal disease, facial nerve dehiscency, incus, and stapes erosion.

## Introduction

Cholesteatoma is a keratin-producing squamous epithelium cyst of the middle ear or mastoid (1). The frequency of cholesteatoma is approximately 42% in chronic otitis media in the Iranian population (2). The most common locations of cholesteatoma are the posterior attic, posterior mesotympanum, and anterior attic; although they may develop anywhere within the pneumatized portions of the temporal bone. Cholesteatoma may be acquired or congenital, with a similar morphologic appearance (3).

The diagnosis of aural cholesteatoma is made simply by otoscopic examination, in addition toendoscopic and microscopic evaluation or even surgical exploration. Special imaging procedures, such as high-resolution computed tomography (HRCT) and magnetic resonance imaging (MRI), may suggest the presence of cholesteatoma within the temporal bone, and may be used to complement the clinical examination (4). Great ossicular erosion is common in cholesteatoma, which results in a poor hearing outcome (5), although CT can not distinguish soft tissue from effusion or granulation tissue inthe patients (6). In attic cholesteatoma, erosion of the scutum (the first sign of aural cholesteatoma) in the coronal view can be assessed clearly. Bony erosion occurs more commonly in the long process of the incus, the body of the incus, and the head of the malleus. Cholesteatoma of the pars tensa extends to the long process of the incus and the superstructure of the stapes. Expansion of aditus ad antrum increases the probability of attic cholesteatoma. The most common site of labyrinthine fistula is the lateral canal dome; and the most involved segment of the facial nerve is the tympanic segment (3). On MRI, cholesteatoma appears with low signal intensity on T1-weighted, and high signal intensity on T2-weighted, images (3). HRCT is the imaging modality of choice for evaluation of middle ear structures and their pathologies such as cholesteatoma (7). An HRCT scan is useful for planning the surgical approach, determining the extension and site of cholesteatoma and its sac, assessing the ossicles, evaluating the facial nerve, tegmen and sinus plate, and determining dural, sigmoid sinus, and jugular bulb positions (8,9). 

CT scan findings of acquired cholesteatoma of the temporal bone consist of a homogenous soft tissue mass with local bone erosion and also middle ear opacification due to granulation tissue, pus, and effusion. Findings suggesting cholesteatoma include attic lateral wall (scutum) erosion , aditus ad antrum widening, dislocation of ossicular chain, erosion of ossicles, labyrinthine fistula, facial nerve canal (fallopian canal) erosion, middle ear and mastoid (tegmen) dehiscence, mastoid destruction (automastoidectomy), sigmoid plate dehiscence, and external auditory canal roof erosion (5). 

In the presence of cholesteatoma, surgical treatment is required except in the elderly or in patients with a poor general medical condition (2). This study assesses the usefulness of a preoperative HRCT scan in depicting the status of middle ear structures in the presence of cholesteatoma. It compares the preoperative temporal bone HRCT findings with intraoperative findings in patients with cholesteatoma.

## Materials and Methods

This cross-sectional study was performed between January 2009 and April 2011 in Al-Zahra and Kashani Hospitals, Isfahan University of Medical Sciences. The study population was 36 cases of aural cholesteatoma who were candidates for surgery. Patients with a history of previous ear surgery, systemic disease which may affect the ear (e.g. collagen vascular or granulomatous diseases), malignancies of the temporal bone and skull base, and those with a history of head and neck radiotherapy were excluded from the study. The study protocol was approved by the ethics committee of Isfahan University of Medical Sciences. 

 A temporal bone HRCT without contrast (axial and coronal view) was performed before surgery in all 36 patients using a Shimatzu 7800TC Computed Tomography Scan in 2 mm cuts, and interpreted by an expert head and neck radiologist. Surgical findings were recorded by the otolaryngologist surgeon. Positive and negative findings, specificity, sensitivity, and accuracy of HRCT were determined by statistical analysis.

 The severity of bone erosion was defined on the basis of CT and surgical findings according to the following scale: one point for scutum erosion, destruction of mastoid air cell network, incus erosion, stapes erosion; Two points for malleus erosion, fallopian canal erosion, widening of aditus ad antrum, external auditory canal (EAC) posterior wall erosion; three points for erosions of sigmoid plate, semicircular canal, tegmen and for intracranial complications. 

The extent of bone destruction was defined as mild (only one-point findings), moderate (at least one two-point finding), or severe (at least one three-point finding). Correlation of the CT scan and surgical findings for bone erosion was determined by the kappa coefficient using SPSS statistical analysis software (version 18). The kappa coefficient ranged from 0–1; with 0 indicating no correlation and 1 indicating complete correlation between the two factors.Kappa coefficients in the range 0–0.4, >0.4–0.6, >0.6–0.8, and >0.8–1.0 indicated poor, moderate, good, and excellent correlation, respectively. 

## Results

Thirty-six patients, including 20 males (55.6%) and 16 females (44.4%), entered the study. The mean age of the patients was 38.2 years. Based on the scoring system used in the study, nine (25%) 22 (61.1%), and 5 (13.9%) patients were categorized as having mild, moderate, and severe disease, respectively. Severe disease was more common among males (80%) (P=0.04) (Table.1).

Positive surgical findings included soft tissue mass in the middle ear or mastoid (100%), ossicular erosion (80%), erosion of the scutum (76%), and aditus ad antrum widening (61%). Positive findings of the temporal bone CT scan included soft tissue mass in the middle ear or mastoid (100%), ossicular erosion (88%), scutum erosion (72%), and aditus ad antrum widening (58%). The greatest precision of CT scan in this study was in detecting sigmoid plate erosion (100%), widening of the aditus ad antrum (97.2%), and scutum erosion (94.4%). 

Table 2 shows the accuracy, sensitivity, specificity, and predictive values for different CT findings. The greatest radiologic-surgical adaptability was in sigmoid plate erosion (kappa=1, P<0.0001), and in widening of the aditus (kappa=0.92, P<0.0001) (Table.2). 

The poorest radiologic-surgical adaptability was in fallopian canal erosion (kappa=0.2, P=0.116), with stapes erosion in second place (kappa=0.27, P=0.091) (Table. 2). Radiologic-surgical adaptability according to the Kendall coefficient was perfect for severe disease (0.84) but was poor for moderate disease (0.34) and fair for mild disease (0.4). There was also a correlation between the surgical finding of erosion of the fallopian canal and the imaging finding of the lateral canal fistula (LCF) in our study (P=0.001) (Table. 3). We also found a correlation between the CT finding of the scutum erosion and the surgical finding of malleus erosion (P=0.002) (Table. 3).

**Table 1:** Severity of disease by surgical and radiological finding

		**CT scan findings**			
		**Mild**	**Moderate**	**Severe**	**Total**
Surgical findings	Mild	8 (88.9%)	1 (11.1%)		9 (100%)
	Moderate		18 (81.8%)	4 (18.2%)	22 (100%)
	Severe			5 (100%)	5 (100%)
	Total	8 (22.2%)	19 (52.8%)	9 (25%)	36 (100%)

**Table 2:** Preoperative high-resolution temporal bone CT scan (axial and coronal view) compared with intraoperative findings

**HRCT findings**	**Accuracy (%)**	**Sensitivity (%)**	**Specificity (%)**	**Positive predictive value (%)**	**Negative predictive value (%)**	**Kappa coefficient**	**P-value**
Sigmoid plate erosion	100	100	100	100	100	1	>0.0001
Aditus expansion	97.22	100	88.8	96.42	100	0.92	>0.0001
Scutum erosion	94.44	96.4	87.5	96.42	87.5	0.83	>0.0001
Tegmen erosion	91.66	75	96.9	60	96.77	0.71	>0.0001
Malleus erosion	80.55	82.4	78.9	77.77	83.33	0.61	>0.0001
Mastoid trabeculae erosion	80.55	76.9	82.6	71.42	86.36	0.58	>0.0001
Lateral canal erosion	86.11	75	87.5	42.85	96.55	0.47	>0.003
Incus erosion	86.11	90.6	50	93.54	40	0.36	0.027
Stapes erosion	63.88	61.9	66.7	72.22	55.55	0.27	0.091
							
Facial canal erosion	75	66.7	75.8	20	75.75	0.20	0.116

A number of the surgical findings had a significant correlation with the CT findings. This correlation was determined by cross tables with the Fisher exact test and shown in Table 3.

**Table 3:** Significant correlation between surgical and CT scan findings (P-value).

		**CT scan findings**				
		**Incus erosion**	**Malleus erosion**	**Stapes erosion**	**Lateral canal fistula**	**Facial canal erosion**
Surgical findings	Scutum erosion	0.001	0.002			
	Tegmen erosion				0.002	
	Lateral canal fistula		0.04			0.001
	Facial canal Erosion				0.001	
	malleus erosion					0.095

## Discussion

This study has demonstrated good correlation between temporal bone HRCT scans with surgical findings, particularly in sigmoid plate erosion, aditus widening, and scutum erosion. It has also demonstrated the advantage of a temporal bone CT scan in the detection of tympanic cholesteatoma, mastoid cholesteatoma, and ossicular chain erosion (10). HRCT was also shown to have better diagnostic value in comparison with conventional CT scans (11). 

Although cholesteatoma has been reported to be more prevalent in men in previous studies (12), we found no statistical difference in our study (P>0.05). It is noteworthy to mention that severe disease in men was more prevalent (P=0.04), perhaps due to delay in medical treatment or biopsychosocial characteristics, and demands further study.

Although some CT findings, such as soft tissue mass, ossicular and scutum erosions, were compatible with other studies, it was not possible to detect fallopian canal and stapes erosions by imaging with CT scan as precisely as in other studies.

The radio-surgical correlation was excellent for the sigmoid plate (kappa statistics, k=1), aditus expansion (0.92), and scutum (0.83); good for the tegmen (0.71) and malleus (0.61); moderate for mastoid trabecula (0.58) and semicircular canals (0.47); but poor for the incus (0.36), stapes (0.27), and facial nerve canal (0.2). 

Our findings contrast with previous reports which showed an excellent radio-surgical correlation for the malleus (kappa statistics, k=0.83), stapes (0.94), and semicircular canals (0.8); good for the incus (0.62) and tegmen (0.65); but poor for the facial nerve canal (0.3) (13). 

Rocher and colleagues revealed excellent correlation for the scutum, the horizontal semicircular canal (>0.7), and the tegmen (0.6), and poor correlation for the facial nerve canal (<0.5) (14).

The correlation found between fallopian canal erosion and LCF in this study may alert the surgeon that detection of one may indicate the existence of the other, potentially leading to a decrease in iatrogenic complications.

The minimal accuracy for stapes erosion observed may be the result of the small size of the bone. Previous studies have used 2–3 mm slices of temporal bone CT scan to detect stapes status (11). Some findings with a low specificity due to the partial volume effect can be improved by employing finer cuts of the CT scan. We also experienced low specificity of CT in the detection of incus erosion. False positive results may be due to a partial volume effect and can be improved by fine slices of CT. Unlike Yu, who reported good correlation between facial canal and incus erosions, we found no such close relationship (4,7).

Fuse and colleagues detected coincidence of semicircular canal fistula, with a sensitivity of 66% and specificity of 84% for facial canal dehiscence, in 59 (97%) of the 61 patients (15). This study also has shown significant correlation between fallopian canal erosion and LCF, which is compatible with previous reports (16). HRCT predicted most of the fistulae except for those with very small sizes (17).This correlation may be mostly due to anatomical proximity of these two mentioned structures, and may help the surgeon to clarify probable complications before surgery. 

The significant correspondence between HRCT and clinical findings may lead to better a diagnosis of probable problems before surgery, and improves the success rate of cholesteatoma surgeries. Limitations of HRCT should be considered and improved by newer radiologic modalities. 

## Conclusion

Preoperative CT scan is helpful in relation to diagnosis and decision making for surgery in cases of cholesteatoma and ossicular erosion. CT scan accurately predicts the extent of disease and is helpful for detection of LCF, erosions of dural plate, and ossicular erosions. However, this technique is not able to distinguish between cholesteatoma and mucosal disease, FND, incus and stapes erosions in the early stages. 
